# Narrow Linewidth Distributed Bragg Reflectors Based on InGaN/GaN Laser

**DOI:** 10.3390/mi10080529

**Published:** 2019-08-11

**Authors:** Wuze Xie, Junze Li, Mingle Liao, Zejia Deng, Wenjie Wang, Song Sun

**Affiliations:** 1 Microsystems and Terahertz Research Center, China Academy of Engineering Physics, Chengdu 610200, China; 2 Institute of Electronic Engineering, China Academy of Engineering Physics, Mianyang 621999, China

**Keywords:** distributed Bragg reflectors, gratings, GaN-based lasers, linewidth

## Abstract

A variety of emerging technologies, such as visible light communication systems, require narrow linewidths and easy-to-integrate light sources. Such a requirement could be potentially fulfilled with the distributed Bragg reflector (DBR) lasers, which are also promising for the monolithical integration with other optical components. The InGaN/GaN-based surface etched DBR is designed and optimized using the finite-difference-time-domain (FDTD) method to obtain very narrow-band reflectors that can serve as a wavelength filter. The results reveal that the ultimate reflectivity depends on the grating period and duty ratio of the DBR. Based on the design, the DBR lasers with various duty ratios are fabricated, specifically, the 19th, 13th and 3rd order DBR grating with duty ratio set as 50%/75%/95%. The minimum linewidth could be achieved at 0.45 nm from the 19th order grating with a 75% duty ratio. For comparison, the Fabry–Pérot (F–P) based on the same indium gallium nitride/gallium nitride (InGaN/GaN) epitaxial wafer are fabricated. The full width at half maximum (FWHM) of the DBR laser shrank by 65% compared to that of the conventional F–P laser, which might be helpful in the application of the visible light communication system.

## 1. Introduction

The visible light communication (VLC) has been paid great attention in both the academic and engineering fields for its broad range of applications, including Li–Fi, vehicle to vehicle communication, underwater communication, and information display, among the others. However, some common challenges still exist for VLC [1,2,3,4,5], such as the integration of the VLC system and higher modulation bandwidth of the light source. The wide-bandgap-GaN-based solid-state lighting source has incomparable advantages for the high-speed VLC [6,7,8] due to its small size, excellent beam quality, long lifetime, and good stability. 

The integration of various components on a single chip still poses an enormous challenge to enhance the functionality, speed, efficiency, and robustness of the overall system. Recently, the chip integration of modulators, waveguides, photo-detectors with gallium nitride (GaN) light-emitting diodes (LEDs) on various substrates have been demonstrated for VLC [9,10,11]. Most of the current VLC systems use LEDs, which suffer from low output powers, bad beam convergences, low modulation bandwidths, and wide linewidths. These parasitic drawbacks are due to the light emission nature of the LEDs, which can be effectively overcome with high-performance lasers. Meanwhile, the laser should be integrated easily into the VLC systems.

A promising light source is the Fabry–Pérot (F–P) laser integrated with the surface etched distributed Bragg reflector (DBR) [12,13]. On the one hand, the convolution between the F–P cavity and the DBR grating selects the longitudinal modes, which is capable of obtaining a more stable and narrower laser emission. On the other hand, the DBRs could be simultaneously fabricated with the ridge waveguide, yielding a much simpler fabrication process than the conventional coating process. Altogether, a high performance on-chip integrated optical system could be envisioned with the DBR laser.

Although DBR lasers in the infrared wavelength have been extensively studied [14,15,16,17,18,19,20], there are few reports about the GaN-based DBR lasers in the visible spectrum [21,22,23]. Cho et al. demonstrated the room temperature operation of an electrically injected InGaN/GaN-based DBR laser and reduced the threshold current density [24]. Wang et al. observed a 50% reduction in the threshold pumping intensity by introducing the DBRs at the end of an InGaN/GaN-based multiple quantum well (MQW) laser [12]. The carrier lifetime and optical bistability in the InGaN-based DBR lasers were reported by Dorsaz et al. [25]. The influence of the surface etched DBRs was studied, for instance, the tilt of the sidewall [26,27]. While more attention is focused on the threshold conditions, the characteristics of the emission linewidth of the GaN-based laser with DBRs have not been thoroughly investigated, which is crucial for the high-speed VLC application.

In this work, the etched surface grating is designed and optimized for the InGaN/GaN-based DBR lasers using the finite-difference-time-domain (FDTD) method. Based on the designed structure, the DBR lasers with different-orders and various duty ratios are fabricated and analyzed to obtain a better light source. The uniform grating structures are defined by electron-beam lithography (EBL) and etched by inductively coupled plasma (ICP). In addition, the conventional F–P laser is also fabricated on the same wafer as the reference sample. The slope efficiency and threshold current for the GaN-based DBR lasers and F–P are compared. Furthermore, the linewidth of the GaN-based lasers with and without the DBR structure is illustrated. 

## 2. Simulation and Fabrication

**Figure 1:**
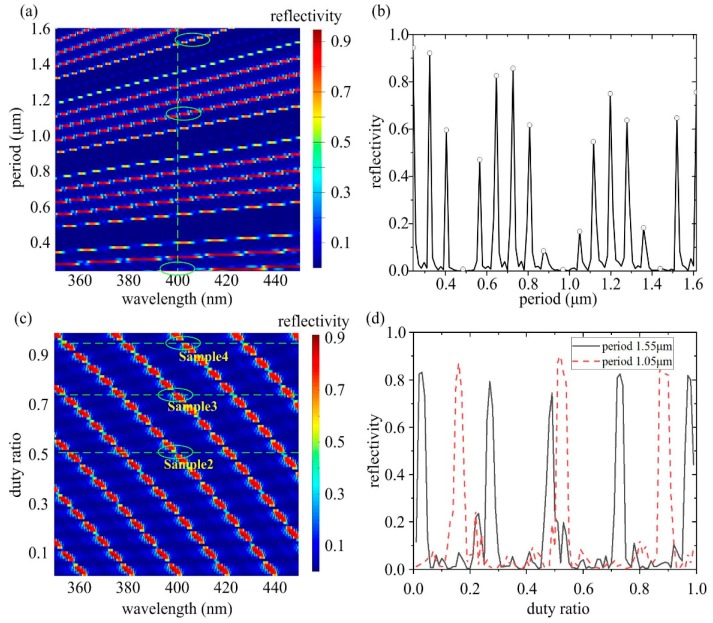
Simulation results of reflection spectra of varied period (**a**) and duty ratio (**c**). (**b**,**d**) are the reflectivity at 400 nm corresponding to (**a**) and (**c**), respectively. For comparison, period of 1050 nm is also plotted in (**d**).

The FDTD simulation is carried out to design the DBR, as shown in Figure 1. To mimic the experimental condition, the grating is assumed to consist of rectangular-shaped grooves. The etched grooves are covered by air (*n*_air_ = 1) and the grating pair number is fixed at 15, which is sufficient to provide high reflectivity with moderate computing resources. According to the Bragg condition *λ_0_ = 2n_eff_Λ/m*, for a given etch depth *d* = 800 nm, the lasing wavelength *λ*_0_ varied with *Λ* and the duty ratio *D* = *(Λ−o)/Λ*, where *n*_eff_ is the longitudinally averaged effective refractive index of the structure, *Λ* is the grating period, *m* is the order of the Bragg grating, and *o* is the width of the etched grooves. In Figure 1, it shows the dependence of reflectivity on *Λ* and *D*, respectively. In Figure 1a,b, the duty ratio is selected at *D* = 80%, the grating period *Λ* varies from 0.24 μm to 1.6 μm. In Figure 1c,d, the grating period is fixed at *Λ* = 1.6 μm, and the duty ratio *D* varies from 0 to 1.0. For comparison, the result for a period of 1050 nm is also plotted in Figure 1d.

It can be seen from Figure 1a,b that the center wavelength of 400 nm could be obtained for an appropriate pair of *Λ* and *D*. A larger maxima reflectivity could be obtained with a shorter grating period, indicating smaller loss. It is noteworthy that the scattering loss at the rough sidewalls is not included in the simulation and it is expected to be more critical for the short period grating. From this point of view, the shorter period DBR does not necessarily have a better reflection performance. On the contrary, the higher-order grating has a narrower reflection bandwidth [28], which is favorable in the communication systems. Combining the above rules, the grating periods of 0.24 μm, 1.05 μm, and 1.55 μm are chosen to obtain various orders of DBR at a specific duty ratio. The desired values are denoted as circles in Figure 1a.

It shows in Figure 1c that the duty ratio has less effect on the maximum reflectivity and the bandwidth of the same order. However, the fabrication tolerance decreases as the grating period increases, since in a long periodic grating, the peak reflectivity has a lower duty ratio tolerance, as shown in Figure 1d. An alternation in the duty ratio actually implies the most common fabrication errors, since a small change in the dose of the e-beam writing process would result in the change in the line/space width, instead of the grating period. The optimized duty ratios of 50%, 75%, and 95% at the period 1.55 μm are set to demonstrate their characteristics. In brief, careful balance between the loss, bandwidth, and fabrication tolerance should be made to achieve the appropriate performances. In the following, the DBR gratings on one end of the F–P cavity with the optimized periods and duty ratios are fabricated and tested to obtain the narrow linewidth lasers. 

**Figure 2:**
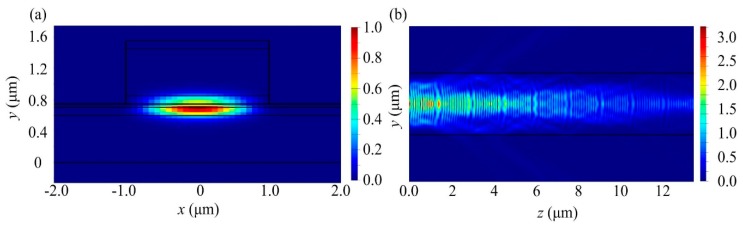
Optical power intensity profile of optimized distributed Bragg reflector (DBR) grating. (**a**) Cross-sectional view; (**b**) top-section view.

The top and cross-section views of the intensity profile of the optimized DBR are shown in Figure 2, where the grating period is 1.55 μm and the duty ratio is 75%. It could be observed that the optical field is confined in the waveguide, and is gradually decayed along the transmission direction, indicating that the optical field is effectively reflected.

**Figure 3:**
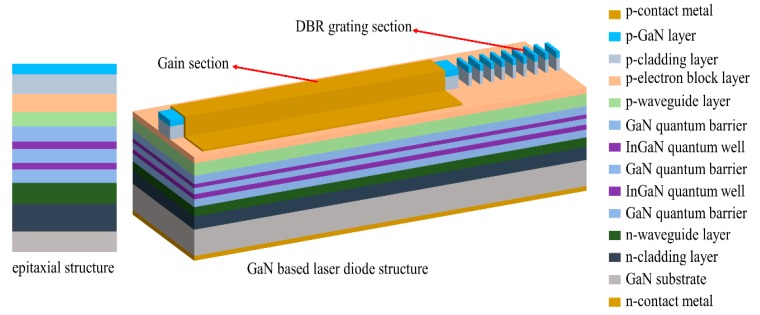
Schematic cross-section of gallium nitride (GaN)-based epitaxial wafer and structure of fabricated DBR laser diode.

The complete structure of the GaN laser diode integrated with the DBR is shown in Figure 3. The sample is first processed into a 10 μm wide ridge structure including an 800 μm length gain section and the DBR section, with the ridge etching stopping at the p-electron block layer by the ion beam etching. Before the deposition of the p-contact metal, a 200 nm thick SiO_2_ layer is deposited on the side of the ridge in order to confine the injection section on the top of the ridge and prevent additional optical loss from the metal contact. Aiming to achieve an emission wavelength at 400 nm, a period of 1550 nm is chosen for the 19th order Bragg grating containing 100 pairs of gratings. This period grating is large enough, and could be precisely defined over the pre-etched DBR section via the electron beam lithography. Followed by the inductively coupled plasma (ICP) etch, the grating structure could be simultaneously transferred into the epitaxial layer. A 10/20/30 nm Pd/Ni/Au p-ohmic contact metal is deposited on the top of the ridge with a 15/300 nm Ti/Au contact pad deposited on the top of it. The GaN substrate is thinned to 100 μm thickness by the chemical mechanical polish method. A 50/50/200 nm Ti/Pt/Au n-contact metal is deposited on the back of the GaN substrate. Finally, the sample is cleaved to form the laser facet, which is perpendicular to the ridge structure. 

The morphologies of the samples are measured by the scanning electron microscope (SEM) with the FEI Nova NanoSEM 450, and the resolution is 1.4 nm at the high-vacuum mode. The power-current-voltage measurement is characterized under the pulse driving condition with a 1 μs pulse width and 10 kHz repetition rate. The spectral characteristics are measured by a fiber spectrometer (BIM6002, Brolight, Hangzhou, China) with a resolution 0.16 nm under the pulse driving condition with a 500 ns pulse width and 1 kHz repetition rate.

## 3. Results

The DBR grating structures of the fabricated laser diodes are shown in Table 1. An F–P ridge waveguide laser diode is set as the blank reference sample named as Sample 1. The DBR grating laser diodes with duty ratios of 50%, 75%, 95%, are denoted as Sample 2, Sample 3, and Sample 4, respectively. All the samples are processed from the same epitaxial wafer and share the same fabrication procedure. The fabricated structures of the DBR grating show little deviations from the designed structure, which is attributed to the slight alternation in the dose of the e-beam writing process.

**Figure 4:**
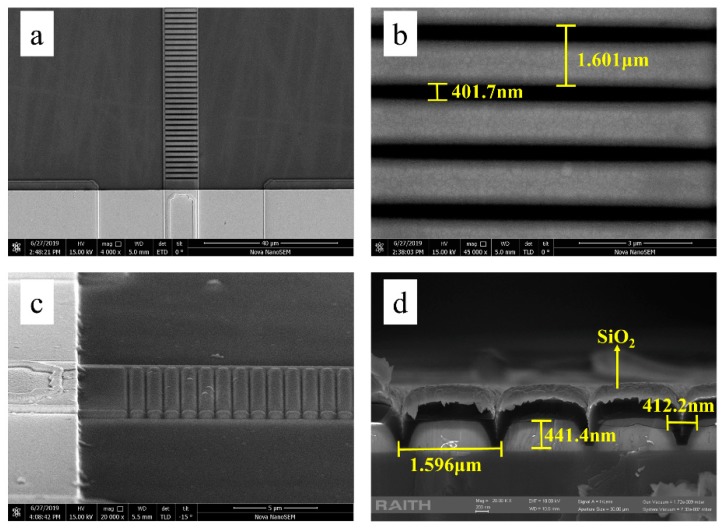
Scanning electron microscope (SEM) imagine of top and side view of 19th order, 75% duty ratio DBR grating fabricated by electron-beam lithography (EBL) and inductively coupled plasma (ICP) dry etch. (**a**) top view; (**b**) top view with high resolution; (**c**) bird view; (**d**) cross section view.

Figure 4 shows the scanning electron microscopy (SEM) picture of a typical dry-etched DBR grating of Sample 4. Obviously, the grating is precisely fabricated over the desired DBR section at the end of ridge waveguide of the laser diode, as shown in Figure 4a. The period and duty ratio of DBR grating are shown in Figure 4b. There is a slight deviation in the grating period and duty ratio between the designed structure and the fabricated device. However, such a small deviation wouldn’t affect the performance of the device. In order to characterize the depth of the device, the DBR grating is cleaved along the ridge waveguide direction. Figure 4c,d show the sidewall and the corresponding sectional view of the DBR grating. The sidewall of DBR grating structure looks smooth, and the depth of the DBR grating is 414.4 nm, which again proves that the devices are well fabricated.

**Figure 5:**
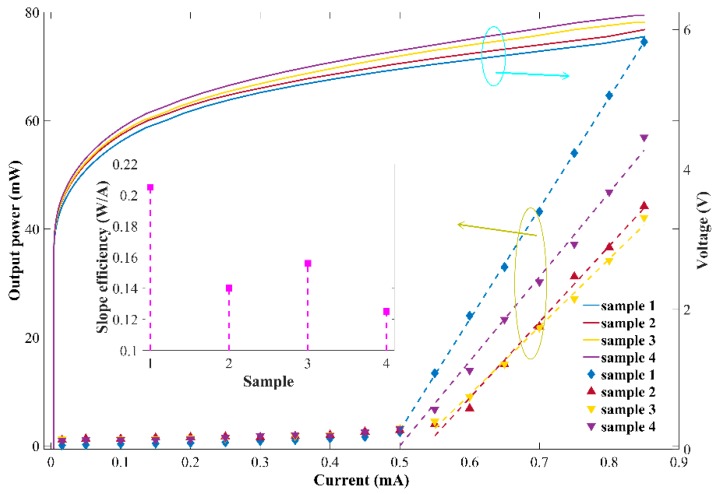
Power-current-voltage (P-I-V) measurement and slope efficiency of Sample 1, Sample 2, Sample 3, and Sample 4 that are characterized under pulse driving conditions, 1 μs pulse width, and 10 kHz repetition rate.

Figure 5 shows the power-current-voltage (P-I-V) characteristics of all the samples. The inset in Figure 5 shows the slope efficiencies of the four samples. The current through the ridge waveguide varies from 10 mA to 850 mA with a step of 50 mA. The threshold current (*I*th) is 490 mA, 540 mA, 530 mA, and 500 mA for the F–P laser diode and three DBR lasers, respectively. All the samples demonstrate similarly high-threshold currents around 500 mA, which is attributed to the imperfect qualities of the epitaxial materials. Another reason could be that the grating is not etched deep enough to provide high reflectivity as desired. The slope efficiency is 0.205 W/A, 0.14 W/A, 0.125 W/A, and 0.156 W/A for the four samples, respectively. It is obvious that the slope efficiency of the F–P laser diode is higher than that of the DBR laser diodes, which is attributed to the diffraction and scattering losses from the gratings. It shares a similar level of the slope efficiency among the DBR laser diodes, while Sample 4 (with a period of 1601 nm of the 19th order gratings DBR laser) shows a relatively higher slope efficiency compared to other counterparts, indicating a smoother sidewall and smaller scattering loss and diffraction loss. It could also be speculated from the current-voltage (I-V) curve that the F–P laser possesses a lower threshold voltage, which is confirmed by the loss of reflectivity in the DBR in the above simulations.

**Figure 6:**
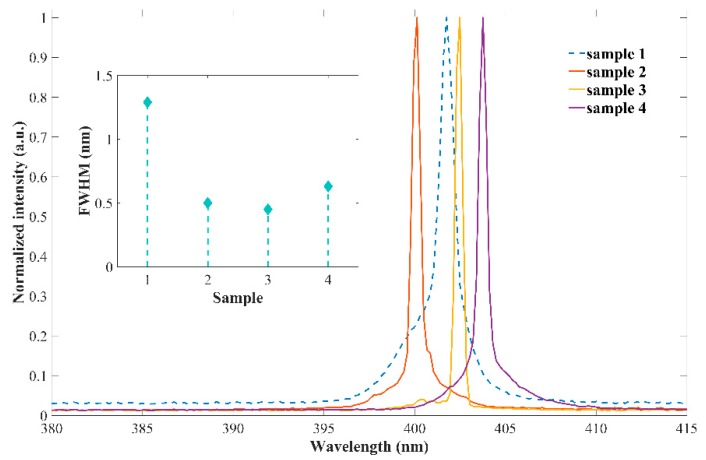
Emission spectra fabricated laser diodes (Sample 1 to Sample 4) under pulse driving conditions with 500 ns pulse width, 1 kHz repetition rate.

The emission spectra of the F–P laser diode and DBR laser diodes (Sample 1 to Sample 4) are characterized at a driving current of 1.5 times that the threshold current (1.5*I*th) by a fiber optic spectrometer with a spectrum resolution of 0.16 nm, and is shown in Figure 6. The emission wavelengths of all samples show slight fluctuations around 400 nm which could be attributed to the inhomogeneity of the epitaxial structure. The full width at half maxima (FWHM) of samples are 1.29 nm, 0.50 nm, 0.45 nm, and 0.63 nm, respectively. It is obvious that the FWHMs of the DBR laser diodes show more than 65% reductions compared to that of the F–P laser, due to the mode selection realized by the DBR gratings. The DBR laser diode with the 75% duty ratio (Sample 3) shows the narrowest line width of 0.45 nm.

**Figure 7:**
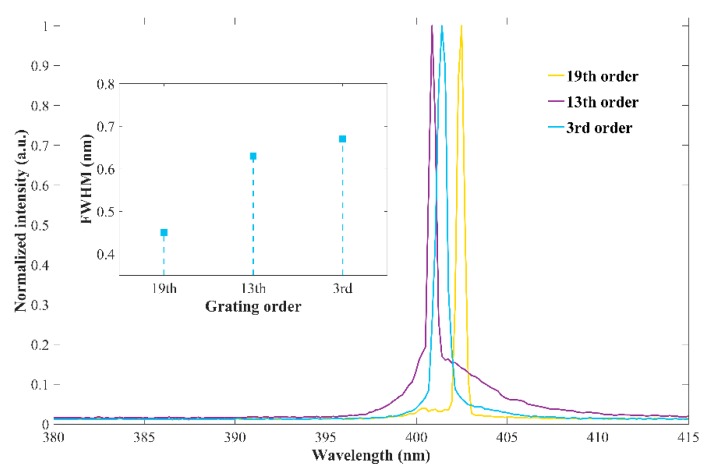
Emission spectrum property of fabricated DBR laser diodes under pulse driving condition with 500 ns pulse width, 1 kHz repetition rate.

The DBR laser diodes with different periods are chosen for the Bragg grating structures to study the influence of the period on the emission spectra. The periods of 1550 nm, 1050 nm, and 240 nm are chosen for the 19th, 9th and 3rd order DBR gratings with the same duty ratio of 75%. Figure 7 shows the emission spectra of the four samples under the pulse driving condition with the injection current of 1.5*I*_th_. It is obvious that the line width of the emission spectrum increases with the decrease of the DBR grating period, which is consistent with the simulation results. In addition, it is easier to fabricate a high-quality DBR grating for a large period, because the structure fluctuation of a large period grating could be easily controlled during the grating manufacture procedure. 

## 4. Conclusions

In summary, the InGaN/GaN-based laser diode integrated with the surface etched DBR grating is designed and optimized using the FDTD method. On top of that, the DBR grating with even and the smooth sidewall is fabricated by the EBL and ICP dry etch, fulfilling the designed structure in the FDTD simulation. The optical and electrical properties are characterized among the DBR laser diodes. About 65% reduction on the emission linewidth can be achieved with a DBR laser diode of 1601 nm period and 75% duty ratio, compared to that of a conventional F–P laser diode. However, the slope efficiency of the DBR laser is lower than the F–P laser because of the scattering losses and absorption loss in the grating. Moreover, the InGaN/GaN-based DBR lasers with 19th, 13th and 3rd, 75% duty ratios are fabricated to investigate the influence of the grating period on the emission linewidth. A minimum linewidth of 0.45 nm could be obtained with a DBR laser diode of 19th order and 75% duty ratio, due to the minimized fabrication tolerance at large scale in the manufacturing procedure. 

## Figures and Tables

**Table 1:** The structure of the fabricated distributed Bragg reflector (DBR) grating.

Sample Name	Sample 1	Sample 2	Sample 3	Sample 4
Designed period	-	1550 nm	1550 nm	1550 nm
Designed duty ratio	-	50%	75%	95%
Fabricated period	-	1583 nm	1601 nm	1589 nm
Duty ratio	-	44%	75%	92%
